# Improvement of Mechanical and Self-Healing Properties for Polymethacrylate Derivatives Containing Maleimide Modified Graphene Oxide

**DOI:** 10.3390/polym12030603

**Published:** 2020-03-06

**Authors:** Won-Ji Lee, Sang-Ho Cha

**Affiliations:** Department of Chemical Engineering Kyonggi University, 154-42, Gwanggyosan-ro, Yeongtong-gu, Suwon, Gyeonggi 16227, Korea; lwj7159@gmail.com

**Keywords:** self-healing, modified GO, nanocomposite, Diels-Alder reaction

## Abstract

In this paper, a self-healable nanocomposite based on the Diels-Alder reaction is developed. A graphene-based nanofiller is introduced to improve the self-healing efficiency, as well as the mechanical properties of the nanocomposite. Graphene oxide (GO) is modified with maleimide functional groups, and the maleimide-modified GO (mGO) enhanced the compatibility of the polymer matrix and nanofiller. The tensile strength of the nanocomposite containing 0.030 wt% mGO is improved by 172%, compared to that of a polymer film incorporating both furan-functionalized polymer and bismaleimide without any nanofiller. Moreover, maleimide groups of the surface on mGO participate in the Diels-Alder reaction, which improves the self-healing efficiency. The mechanical and self-healing properties are significantly improved by using a small amount of mGO.

## 1. Introduction

Polymers have been used in various applications, such as biomedical devices, transportation, artificial electron skin and coating [1,2,3,4]. However, polymers are vulnerable to damage and loss of durability to external stimuli; thus, polymer composites have been widely studied as a solution to these problems [5]. They have been used in a variety of fields, owing to their superior properties such as good abrasion resistance, corrosion resistance, improved mechanical properties and a light weight. 

Graphene-based materials have outstanding properties such as high conductivity, thermal stability, good mechanical properties and compatibility with polymers [6,7,8]. The durability of polymer composites depends on the integrity of the polymer matrix and reinforcing material such as nanofiller [9]. Poor compatibility between the polymer matrix and the filler causes micro cracks. Thus, the durability and interfacial adhesion are improved by the surface treatment of the reinforcing material [10]. The fillers used in polymer composites are modified by various methods [11] to reduce aggregation, which degrade the mechanical properties—however, these forms of damage are inevitable.

Self-healing, in which the original properties of the material are recovered, is applied to increase the lifetime and durability of these materials. Self-healing materials can be classified into two categories: extrinsic and intrinsic self-healing. One category is extrinsic self-healing using microcapsules, including healing agents. Under an external stimulus, the healing agents in microcapsules are applied to crack locations, and help prevent crack propagation [12]. The other category is intrinsic self-healing, which inherently restores its original properties using stimuli such as heat, light and microwaves [13,14,15,16,17,18,19]. Among intrinsic self-healing, there is much research about the Diels-Alder reaction, which is applicable under mild conditions [20,21]. The Diels-Alder reaction can be applied to various polymers. Most of all, there are many studies that apply the Diels-Alder reaction to acrylate polymer, which is widely used in coating materials [22,23,24,25,26,27,28,29]. In particular, the application of graphene or modified graphene oxide to self-healing polymers not only improves their mechanical properties, but also their healing efficiency [30,31,32,33,34,35,36,37].

In this study, a polymer containing furan groups was designed to apply a self-healing system based on the Diels-Alder reaction. Furthermore, graphene oxide (GO) with excellent mechanical properties was applied as a filler. To improve the dispersity of the filler and the degree of the Diels-Alder reaction, graphene oxide modified with a maleimide moiety (mGO) was prepared, and nanocomposites with different mGO contents were fabricated. The mechanical properties and self-healing efficiency were investigated by tensile testing. Additionally, to confirm the effects of the mGO, a nanocomposite incorporating GO as a control sample was prepared, and compared with the nanocomposite containing mGO.

## 2. Materials and Methods 

### 2.1. Materials

Furan, maleic anhydride, 2-furoyl chloride (FC) and 2-aminoethanol were purchased by Tokyo Chemical Industry Co., Ltd, Tokyo, Japan. Dibutyltin dilaurate (DBTDL), hexamethylene diisocyanate (HDI), graphite, 2-hydroxyethyl methacrylate (HEMA), poly (ethylene glycol) methyl ether methacrylate Mn300 (PEGMMA) and 1,1′-(methylenedi-4,1-phenylene)bismaleimide (BM) were purchased from Sigma-Aldrich Korea Ltd, Yong-In, Korea. For the solvent, tetrahydrofuran (THF), methylene chloride (MC), *n*-hexane, chloroform, *N*,*N*′-dimethylformamide (DMF), methanol and toluene were purchased from Daejung Chemical & Materials Co., Ltd, Si-Heung, Korea. All of these materials were used as received. Azobisisobutyronitrile (AIBN) from Junsei Chemical Co., Ltd, Chuo-ku, Tokyo was used after the recrystallization process. 

### 2.2. Preparation of Maleimide Modified GO (mGO)

mGO was prepared as follows. First, to prepare protected maleic anhydride (PM), maleic anhydride and furan were dissolved in toluene, stirred at 80 °C for 24 h and cooled to room temperature. As the solution cooled, a white solid was obtained. The crystallized white solid was filtered under reduced pressure, washed with a sufficient amount of methanol and dried overnight in a vacuum. Secondly, protected 2-hydroxyethyl maleimide (PHEM) was prepared. PM dissolved in methanol was placed in a two-necked flask, and 2-aminoethanol dissolved in methanol was dropped into this solution. In this process, a round bottom flask was placed in an ice water bath. After all the reactants were completely dissolved in the solvent, the reaction proceeded under reflux conditions (approximately 105 °C) for 24 h. After the reaction, the solution was cooled, and a yellow solid was obtained. Next, the PHEM was deprotected by refluxing in toluene overnight. The solution was then cooled in a freezer to recrystallize the deprotected 2-hydroxyethyl maleimide (HEM) in the form of a white solid. This solid was filtered by vacuum filtration to remove unwanted reactants. Finally, NCO-functionalized maleimide (NCO-M) was synthesized. The HEM obtained by the previous synthesis was dissolved in chloroform. This solution was dropped into a solution containing excessive HDI, and then a few drops of DBTDL were added very slowly. After the drops were added, the reaction proceeded overnight. The resulting solution was poured into excessive *n*-hexane and the precipitate was filtered and dried under reduced pressure. To obtain mGO, the NCO-M and GO prepared by the modified Hummer’s method [38] were diluted in DMF. To improve the dispersion of the GO, this mixture was sonicated for 30 min at room temperature. After sonication, a few drops of DBTDL were added to the dispersed solution. The reaction proceeded for 24 h at 55 °C, and the dispersed solids were filtered using vacuum filtration. The final products were washed with chloroform several times to remove unreacted NCO-functionalized maleimide, and then dried in a vacuum oven. 

### 2.3. Preparation of Furan Functionalized Polymethacrylate

The furan-functionalized methacrylate (FEEMA) monomer was synthesized using our previously reported methods [39]. Subsequently, a series of copolymers with different furan side group contents were prepared by free radical polymerization using FEEMA and PEGMMA. A mixture of FEEMA, PEGMMA and AIBN was stirred under reflux conditions in THF. Next, the mixture was purified twice by precipitation using *n*-hexane. The copolymers are labeled FEEMA#, where # indicates the molar feed ratio of FEEMA and PEGMMA. For example, the copolymer FEEMA64 has a molar ratio of FEEMA:PEGMMA = 6:4. 

### 2.4. Preparation of Self-Healable Furan-Functionalized Polymethacrylate Nanocomposite Films 

Nanocomposite films containing nanofillers, FEEMA64 and BM were fabricated as follows. A selected amount of the nanofillers (mGO or GO) was added to a DMF solution containing FEEMA64 and BM. After sonication at room temperature for 1 h, most of the DMF was removed using a rotary evaporator. The concentrated solution was poured onto silicone substrates, and the remaining DMF was evaporated for 36 h at 90 °C. Subsequently, the retro Diels-Alder reaction proceeded for 6 h at 120 °C, while the Diels-Alder reaction proceeded for 24 h at 50 °C, yielding nanocomposite films. A polymer film without nanofillers was also prepared using the above procedure. Nanocomposite films containing 0.015, 0.030 and 0.050 wt% mGO are labeled FEEMA64_mGO0.015 wt%, FEEMA64_mGO0.030 wt% and FEEMA64_mGO0.050 wt%, respectively. The control samples containing GO were labeled FEEMA64_GO0.015 wt%, FEEMA64_GO0.030 wt% and FEEMA64_GO0.050 wt%, respectively. The polymer film without a filler is referred to as the FEEMA64 polymer film.

### 2.5. Characterization Methods

Fourier-transform infrared (FT-IR) analysis was conducted at 400–4000 cm^−1^ using the Alpha-Platinum attenuated total reflectance model from Bruker, Seongnam-si, Korea. The thermal properties were measured using thermogravimetric analysis (TGA) and differential scanning calorimetry (DSC). For TGA, the TGA 4000 from Perkin Elmer, Seoul, Korea was used under a N_2_ atmosphere. The sample was heated from 40 °C to 700 °C at a rate of 10 °C/min. For DSC, the Discovery DSC 25 from TA Instruments, Seoul, Korea) was used under a nitrogen atmosphere from -40 °C to 180 °C, at a heating rate of 10 °C/min. Optical microscopy (OM) DIMIS-M Siwon Optical Technology Anyang-si, Korea) with a 100× lens was used for surface analysis. The mechanical properties were characterized using a Qmesys universal testing machine (UTM, QM100S, Uiwang-si, Korea) at a crosshead speed of 10mm/minute. The sample dimensions were 15 mm × 50 mm × 0.9 mm. The morphology was analyzed by field emission scanning electron microscopy (FE-SEM, JSM-760F PLUS, JEOL, Seoul, Korea). Transmission electron microscopy (TEM, Tecnai G2 F30 from FEI Company, Suwon-si, Korea) was performed at 300 kV. X-ray diffraction (XRD, Empyrean, Malvern PANalytical, Seongnam-si, Korea) patterns were obtained using Cu Kα radiation at room temperature. Elemental analysis was performed using Flash EA 1112 from Thermo Fisher Scientific, Waltham, MA US. 

## 3. Results

### 3.1. Synthesis and Characterization of mGO

**Scheme 1:**
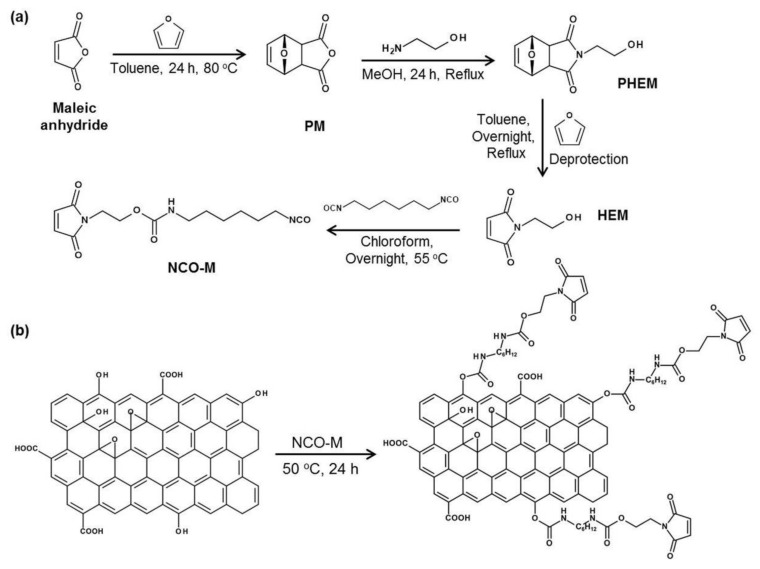
Synthesis of (**a**) NCO-M and (**b**) mGO.

GO obtained via the modified Hummer’s method was modified by maleimide functional groups to act as an analogous healing agent. In addition, mGO is expected to improve the mechanical properties of the polymer matrix. For mGO synthesis, NCO-M was prepared as follows. First, the protecting reaction of maleic anhydride was proceeded to prevent the side reaction during subsequent synthetic processes, such as the maleic anhydride ring-opening reaction. The protecting process was performed via the Diels-Alder reaction of maleic anhydride and furan without any catalyst. After this protecting process, the reaction between PM and 2-ethanolamine was conducted. Next, deprotection was performed via the retro Diels-Alder reaction under reflux conditions overnight, yielding HEM. Finally, the NCO-functionalized maleimide, NCO-M, was obtained via the urethane reaction between the hydroxyl group of HEM and the isocyanate group of HDI, where an excessive amount of HDI was used to prevent the production of bismaleimide. The overall process of NCO-M preparation is shown in Scheme 1a.

The mGO was obtained in the presence of DBTDL, which acted as a catalyst to enhance the reaction between the isocyanate group of NCO-M and the hydroxyl group of the GO obtained using the modified Hummer’s method. Before the reaction, both GO and NCO-M diluted with DMF were sonicated for 30 min to obtain a well-dispersed solution. After the DBTDL catalyst was added, the reaction proceeded for 24 h at 55 °C. The procedure for mGO preparation is shown in Scheme 1b. 

**Figure 1:**
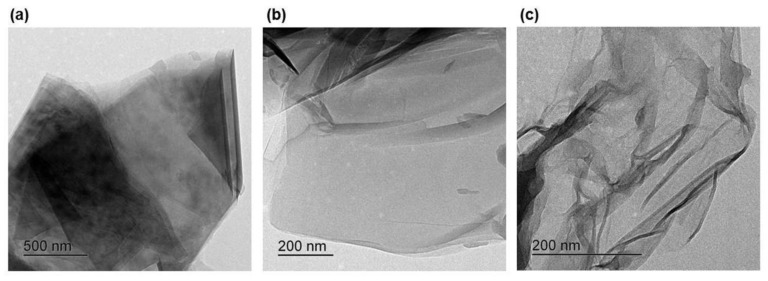
TEM images of (**a**) graphite, (**b**) GO and (**c**) mGO.

The morphologies of graphite, GO and mGO were observed using TEM, as shown in Figure 1. The graphite had multiple layers, but both the GO obtained via the modified Hummer’s method and the mGO had a single layer, which was in agreement with many previous observations. Although some rough and curled edges appeared in the mGO but not in the GO, the modification of the GO caused almost no significant morphological changes.

**Figure 2:**
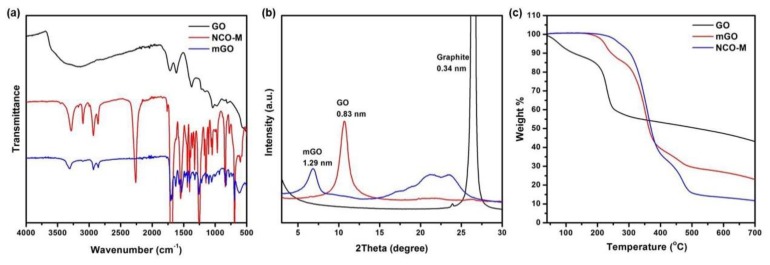
(**a**) FT-IR spectra of GO, NCO-M and mGO, (**b**) XRD patterns of graphite, GO and mGO and (**c**) TGA curves of GO, NCO-M and mGO.

The chemical modification of GO to mGO is confirmed via FT-IR and XRD analyses. Figure 2a shows the FT-IR spectra of NCO-M, GO and mGO. The presence of characteristic peaks of GO, such as the hydroxyl (3000–3700 cm^−1^), carbonyl (1730 cm^−1^), epoxy (1217 cm^−1^) and alkoxy (1056 cm^−1^) groups, confirmed that the oxidation reaction occurred [40,41]. In the mGO spectrum, all the signature peaks of NCO-M are also observed, except the characteristic peak at 2260 cm^−1^, which originates from the isocyanate group in NCO-M [42], indicating that GO was successfully modified to mGO via the urethane reaction between GO and NCO-M. The modification of GO by NCO-M was further confirmed by the XRD patterns of graphite, GO and mGO, as shown in Figure 2b. The interlayer spacing increases in the order of graphite, GO and mGO. The interlayer spacing of graphite calculated by Bragg’s law at the sharp characteristic diffraction peak 26.530° was 0.34 nm; however, GO is found to have an interlayer spacing of 0.83 nm from the 10.675° peak [43]. The fact that GO has a larger interlayer spacing than graphite is attributed to the presence of various oxygen-containing groups on the surface of GO [44]. The interlayer spacing of mGO increased further to 1.29 nm, indicating that the relatively large molecular sized NCO-M was successfully introduced into the surface of GO. The broad peak at approximately 23.668° in the XRD pattern of mGO might be due to the formation of more closely stacked nanosheets, resulting from a reduction in the number of oxygen-containing groups on GO during modification [45,46].

TGA was performed to determine the grafting ratio of the maleimide functional group in mGO, and investigate the thermal stability (Figure 2c). The initial thermal decomposition of GO occurs below 160 °C by the generation of vapors such as CO, CO_2_ and H_2_O [47,48]. The major thermal decomposition, from 160 °C to 270 °C, is attributed to the decomposition of labile oxygen-containing groups on the surface of GO, such as the hydroxyl and carbonyl groups [49]. In the three-step thermal decomposition of mGO, the first and second thermal decomposition steps are attributed to the vapor generations and the decomposition of unstable functional groups containing oxygen, respectively, which is similar to the behavior of GO. Considering the thermal decomposition behavior of NCO-M, the third thermal decomposition step of mGO, which occurred below 490 °C, should be attributed to the decomposition of NCO-M, which is chemically bonded to the surface of the mGO. The amount of residual mGO at 700 °C is found to be 22%, and the amount of residual of GO is 41% at the same temperature, indicating that 19% of the mGO consists of maleimide functional groups [50].

### 3.2. Preparation of FEEMA# Copolymers

**Scheme 2:**
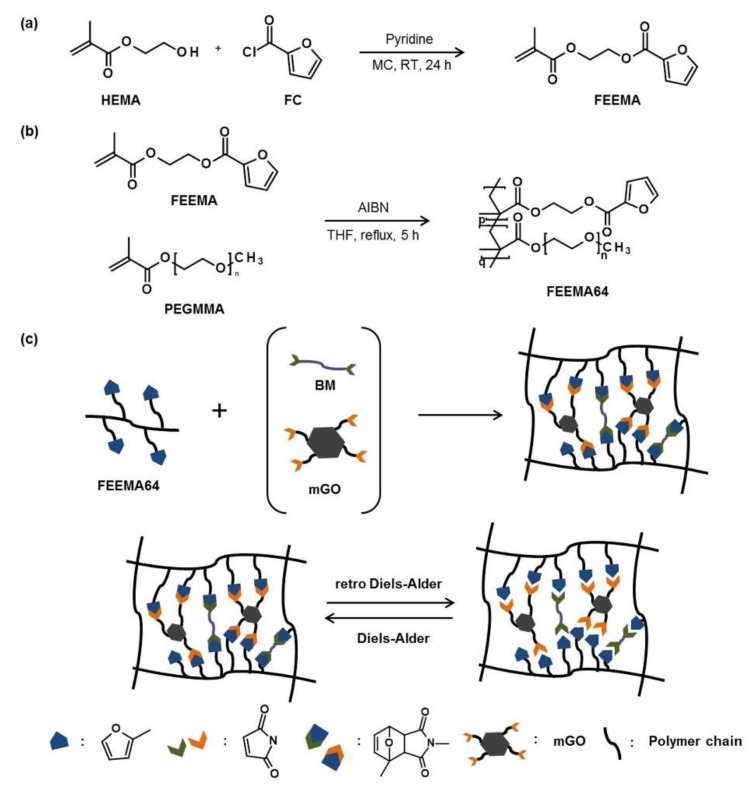
(**a**) Synthesis of FEEMA monomer, (**b**) synthesis of FEEMA64 polymer and (**c**) overall procedure for obtaining nanocomposites.

The synthetic methods of the furan-functionalized methacrylate (FEEMA) and FEEMA# copolymers were described in our previous research, and are illustrated in Scheme 2a,b, respectively [39]. Additionally, the elemental analysis was performed to confirm the chemical composition of FEEMA monomer. Based on the chemical structure of FEEMA monomer, the theoretical value of the C, H and O elements of FEEMA monomer could be expected to reach 39.286 atomic %, 42.857 atomic % and 17.857 atomic %, respectively. It is confirmed that the experimental value of each element obtained from the elemental analysis is almost the same with one of a theoretical value (Table 1). Thus, it is proved that FEEMA monomer has the expected chemical structure. 

The FEEMA/PEGMMA ratio of the copolymer was controlled by changing the feed ratio of each monomer for polymerization. However, a polymer film using FEEMA100 was not obtained, owing to extreme brittleness. Since FEEMA55 had relatively few furan-functional groups, it was considered to be unsuitable for showing the effects of the Diels-Alder reaction in this system. Therefore, FEEMA64 was chosen from among the various FEEMA polymers as a model for further analysis.

### 3.3. Characterization of FEEMA64 Nanocomposites

Scheme 2c illustrates the formation of self-healable nanocomposites using FEEMA64, BM and a filler. As described in Scheme 2c, the furan moiety of FEEMA64 and maleimide moiety of mGO and bismaleimide form self-healable nanocomposites via the Diels-Alder reaction. When damage occurs to the formed nanocomposite and it is heated to a temperature where the retro Diels-Alder reaction can proceed, the Diels-Alder bond (DA bond) dissociates. When the Diels-Alder reaction temperature is applied to the sample again, the healing process take place as the DA bond is recombined. Before the self-healable properties of the nanocomposites are described, the Diels-Alder reaction between the furan-functional group of FEEMA64 and the maleimide group of BM is characterized using FT-IR analyses of the FEEMA64 polymer film.

**Figure 3:**
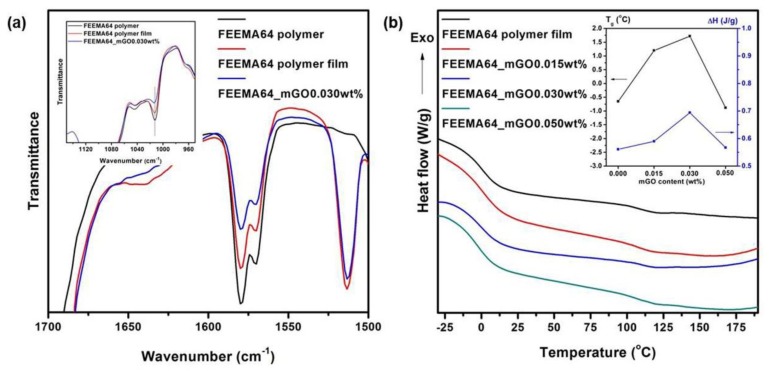
(**a**) FT-IR spectra of FEEMA64 polymer, FEEMA64 polymer film and FEEMA64_mGO0.030 wt%, (inset) furan ring breathing absorption and (**b**) DSC curves of FEEMA64 polymer film and various nanocomposites, (inset) comparison of glass transition temperature and enthalpy at different mGO contents.

As shown in Figure 3a, the characteristic peaks at 1581 and 1569 cm^−1^, which originated from the furan double bond, decreased after mixing FEEMA64 polymer and BM, showing that the Diels-Alder reaction occurred in the FEEMA64 polymer film [51]. Furthermore, in the case of FEEMA64_mGO0.030 wt%, the characteristic peaks attributed to the furan double bond are reduced compared to the polymer film; this was additionally confirmed via the peak at 1014 cm^−1^, attributed to furan ring breathing [52]. As shown in the inset graph of Figure 3a, the peak at 1014 cm^−1^ of FEEMA64_mGO0.030 wt% was lower compared to that of the polymer film. Therefore, it was confirmed that the maleimide-functional groups on the surface of mGO could react with the furan moiety of FEEMA64 polymer via two characteristic peak intensity changes. 

On the basis of the FT-IR observations of the FEEMA64 polymer film, the Diels-Alder reaction in the nanocomposite is verified by DSC analyses, as shown in Figure 3b. Regardless of the filler contents, an endothermic peak is observed at 110°–130 °C, owing to the retro Diels-Alder reaction in the nanocomposite. This behavior indicates that the nanocomposite may also be applicable as a thermally self-healable material [53]. While the glass transition temperature of the polymer film represents −0.650 °C, that of nanocomposites containing mGO increased up to 1.720 °C, as the mGO content of the nanocomposite increased to 0.030 wt%. This is because an mGO existing between polymer chains acts as a reinforcement agent, hindering the segmental motion of the polymer chains as it is already known [54]. Furthermore, as shown in the inset graph of Figure 3b, the variation of the enthalpy depending on the mGO content shows a similar tendency to that of the glass transition temperature. It indicates that the degree of the Diels-Alder reaction increased alongside increasing the maleimide groups on the surface of mGO. [55]. Thus, it could be concluded that mGO has two functions in the nanocomposites. First, mGO acts as a reinforcement agent to enhance mechanical properties, and second, the maleimide-functional groups on the surface of mGO participate in the Diels-Alder reaction to improve healing efficiency. However, in the case of FEEMA64_mGO0.050 wt%, the effect of mGO is reduced due to aggregation. This was further confirmed by the analyses of the mechanical properties of the nanocomposites, as described in the next section.

### 3.4. Mechanical Properties of FEEMA64 Nanocomposite

**Figure 4:**
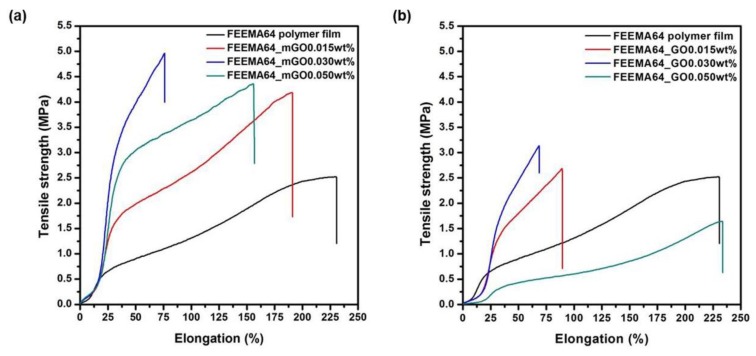
Mechanical properties of nanocomposites containing (**a**) mGO and (**b**) GO.

According to the DSC analyses, the mechanical properties of the nanocomposites can be expected to vary, depending on the mGO content. As shown in Figure 4a, the tensile strength increased with an increasing mGO content of up to 0.030 wt%. Specifically, the tensile strength of FEEMA64_mGO0.030 wt% improved significantly, to reach 4.566 MPa, with an improvement of 172% compared to that of the FEEMA64 polymer film, which contains no nanofiller. However, the tensile strength of FEEMA64_mGO0.050 wt% decreased to 4.022 MPa, exhibiting behavior similar to that of the glass transition temperature in the DSC experiments. The effect of the maleimide groups on the mGO surface was confirmed by a comparison of the tensile strength of the nanocomposites containing GO—that is, the FEEMA64_GO series (Figure 4b). Although the variation in tensile strength change for the FEEMA64_GO series is similar to that of the FEEMA64_mGO series, the absolute tensile strengths values of the entire FEEMA64_GO series are lower than those of the FEEMA64_mGO series. This difference is especially noticeable for nanocomposites with a 0.030 wt% filler content. That is, the tensile strength of FEEMA64_mGO0.030 wt% is 4.566 MPa, while that of FEEMA64_GO0.030 wt% is 3.111 MPa, at an increase of 117% compared to the FEEMA64 polymer film. Therefore, an additional covalent bonding based on the Diels-Alder reaction between the maleimide groups on the surface of mGO and the furan group of FEEMA64 polymer should induce the significantly improved tensile strength of the FEEMA64_mGO series.

**Figure 5:**
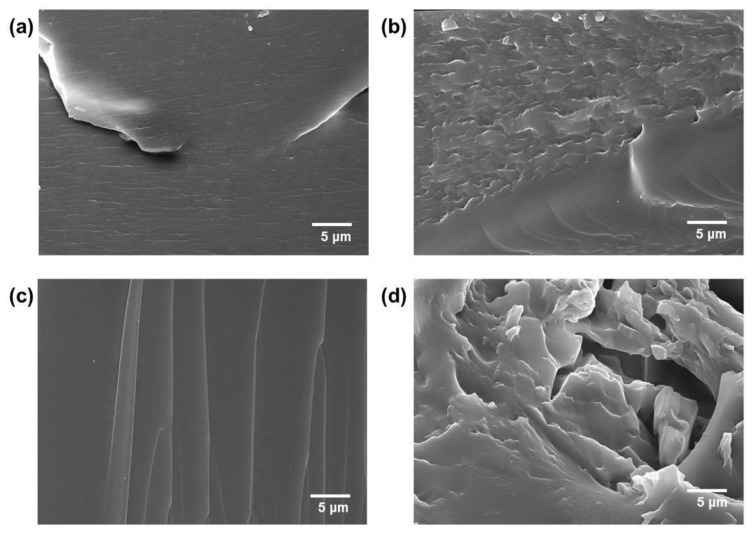
SEM images of (**a**) FEEMA64_mGO0.030 wt%, (**b**) FEEMA64_mGO0.050 wt%, (**c**) FEEMA64_GO0.030 wt% and (**d**) FEEMA64_GO0.050 wt%.

The dispersion of fillers in the nanocomposites is expected to affect their mechanical properties, as has already been discussed [56]. Therefore, the cryogenically fractured surface morphology of FEEMA64_mGO0.030 wt% and FEEMA64_mGO0.050 wt% was compared using SEM images (Figure 5a,b). While a smooth surface which originates from relatively well-dispersed mGO is exhibited in FEEMA64_mGO0.030 wt%, non-homogeneous and rough surface are observed due to the restacking of mGO in the case of FEEMA64_mGO0.050 wt%. This restacking and agglomeration of mGO is expected to decrease the chances of the Diels-Alder reaction between the maleimide groups on the surface of the mGO and the furan groups in the FEEMA64 polymer, meaning the tensile strength of FEEMA64_mGO0.050 wt% should decrease compared to that of FEEMA64_mGO0.030 wt%. A similar tendency with a relatively higher dispersity of GO in FEEMA64_GO0.030 wt% than one in FEEMA64_GO0.050 wt% is also observed in the nanocomposite containing GO, as shown in Figure 5c,d. This implies that the agglomerated GO, which could induce even the formation of a void, disrupts the orientation of the polymer chain in the nanocomposite [57], which in turn relatively degrades mechanical properties of FEEMA64_GO0.050 wt% [58].

### 3.5. Self-Healing Property 

**Figure 6:**
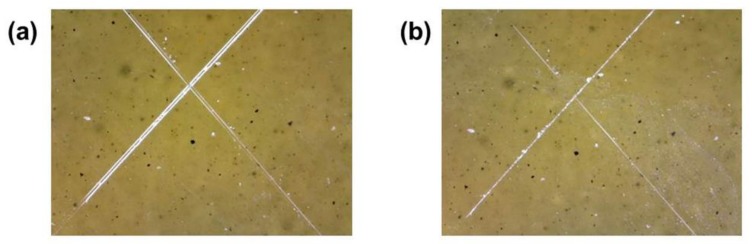
OM images (**a**) before and (**b**) after healing of FEEMA64_mGO0.030 wt%.

First, the self-healing properties of the nanocomposites were investigated by observing the degree of disappearance of surface cracks formed using a razor blade. Figure 6 shows that the optical microscopy images of a FEEMA64_mGO0.030 wt% before and after the self-healing procedure for the retro Diels-Alder reaction at 150 °C for 2 h and the Diels-Alder reaction at 50 °C for 24 h, respectively. Although cracks still appear in some locations, most of the cracks faded or disappeared after the healing procedure. This implies that the polymer chains’ mobility increased sufficiently so that the polymer could penetrate the cracks, and then fill them by the formation of chain entanglements during the retro Diels-Alder reaction.

**Figure 7:**
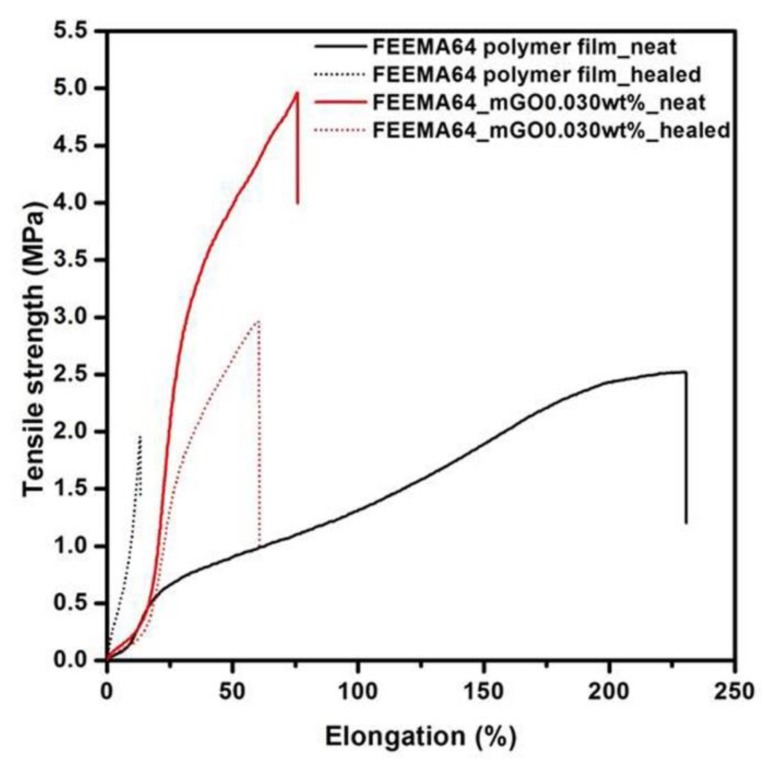
Stress-strain curves of neat and healed samples for FEEMA64 polymer film and FEEMA64_mGO0.030 wt%.

To measure the self-healing efficiency quantitatively, the mechanical properties of the nanocomposites are analyzed using a UTM, as shown in Figure 7. For the healing procedure, an as-prepared neat nanocomposite film is cut using scissors, and the damaged film is then placed on the same silicone mold that was used to fabricate the neat sample. The damaged nanocomposite film, under pressure from the silicone mold, is placed on a hot plate at 150 °C for 2 h. In this process, DA adducts in the polymer matrix were dissociated. After that, the sample was heated at 50 °C for 24 h, which promotes the recombination of DA adducts. The sample obtained by this process is called the healed sample.

The self-healing efficiency is obtained as the ratio of the elongation at break values of the healed samples to those of the neat ones from the UTM experiments [53]. The self-healing efficiency increased gradually with the increasing mGO content of the nanocomposites. In particular, the self-healing efficiency increased dramatically from 23.560% for FEEMA64_mGO0.015 wt% to 81.228% for FEEMA64_mGO0.030 wt%. The self-healing efficiency of FEEMA64_mGO0.050 wt% is similar to that of FEEMA64_mGO0.030 wt%. The self-healing property of the FEEMA64_mGO series is summarized in Table 2. Considering the tensile strength variation of the FEEMA64_mGO series, both the dispersity of mGO in the nanocomposite and the degree of the Diels-Alder reaction between the maleimide groups of the mGO surface and the furan groups in the FEEMA64 polymer should be key factors affecting the self-healing efficiency of the FEEMA64_mGO series; this was further confirmed by the self-healing efficiency of the FEEMA64_GO series. The self-healing efficiency of the FEEMA64_mGO series is much higher than that of the FEEMA64_GO series. For example, the self-healing did not occur in FEEMA64_GO0.050 wt%. However, the self-healing efficiency of FEEMA64_mGO0.050 wt% is 82.033%. The reason for this is thought to be that the maleimide groups on the mGO surface, which participate in the Diels-Alder reaction, improve the self-healing ability of the FEEMA64_mGO series [59]. 

**Figure 8:**
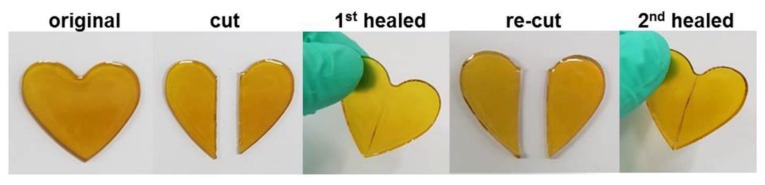
Confirmation of self-healing using FEEMA64_mGO0.030 wt%.

A heart-shaped specimen was prepared to confirm the effect of the reversibility of the Diels-Alder reaction on self-healing property. The healing procedure described above was applied to the heart-shaped specimen, which was cut into two pieces using scissors. The two pieces of FEEMA64_GO0.030 wt% did not bond with each other, indicating that FEEMA64_GO0.030 wt% is not capable of reversible healing. By contrast, as shown in Figure 8, FEEMA64_mGO0.030 wt% was restored to its original shape, even after a second self-healing procedure. This result proves that reversible self-healing, which is a characteristic of the Diels-Alder reaction, is possible for the FEEMA64_mGO specimens. 

## 4. Conclusions

In this report, a furan-functionalized polymer (FEEMA64) was prepared for a polymer matrix. FEEMA64 polymer film was then fabricated using the Diels-Alder reaction between FEEMA64 and bismaleimide. To enhance the mechanical properties of the FEEMA64 polymer film, a graphene-based filler was introduced into polymer film. The graphene-based filler was modified using NCO-M to prevent aggregation. Additionally, the maleimide moiety on the surface of mGO could participate in the Diels-Alder reaction. Thus, the specimens containing mGO exhibited better mechanical properties and self-healing efficiency in this self-healing system. The tensile strength of FEEMA64_mGO0.030 wt% improved to 4.566 MPa, which is an improvement of 172% compared to that of polymer film. The self-healing efficiency of FEEMA64_mGO0.030 wt% is 81.228%, which is significantly better than that of polymer film. Therefore, the mechanical and self-healing properties of brittle polymethacrylate derivatives could be increased by the addition of a very small amount of mGO in this system. This nanocomposite, based on the Diels-Alder reaction, is expected to extend the lifetime of materials by applying smart technology to coating materials. 

## Figures and Tables

**Table 1:** Elemental analysis of FEEMA monomer.

	C (Atomic %)	H (Atomic %)	N (Atomic %)	O (Atomic %)
Theoretical value	39.286	42.857	-	17.857
Experimental value	35.484	48.387	-	16.129

**Table 2:** Self-healing efficiencies and mechanical properties of FEEMA64_mGO series before and after the healing test.

Sample	Before Healing Test		After Healing Test		Self-Healing Efficiency ^1^ (%)
	Tensile Strength (MPa)	Elongation at Break (%)	Tensile Strength (MPa)	Elongation at Break (%)	
FEEMA64 polymer film	2.648 ± 0.257	214.667 ± 14.687	1.663 ± 0.261	12.600 ± 0.898	5.724
FEEMA64_mGO0.015 wt%	4.021 ± 0.229	198.235 ± 10.232	3.611 ± 0.155	36.465 ± 12.070	23.560
FEEMA64_mGO0.030 wt%	4.566 ± 0.564	69.035 ± 9.383	2.916 ± 0.171	61.465 ± 1.322	81.228
FEEMA64_mGO0.050 wt%	4.022 ± 0.542	155.510 ± 6.980	2.927 ± 0.151	141.333 ± 7.588	82.033

^1^ Healing efficiency calculated using elongation at break.
